# Relative Abundances of Species or Sequence Variants Can Be Misleading: Soil Fungal Communities as an Example

**DOI:** 10.3390/microorganisms9030589

**Published:** 2021-03-13

**Authors:** Lukas Beule, Markus Arndt, Petr Karlovsky

**Affiliations:** Molecular Phytopathology and Mycotoxin Research, University of Goettingen, 37077 Goettingen, Germany; markus.arndt@stud.uni-goettingen.de (M.A.); pkarlov@gwdg.de (P.K.)

**Keywords:** temperate agroforestry, tree-based intercropping, alley cropping, soil fungal community, amplicon sequencing, real-time PCR, soil microbiome

## Abstract

Plant production systems that are more sustainable than conventional monoculture croplands are the vision of future agriculture. With numerous environmental benefits, agroforestry is among the most promising alternatives. Although soil fungi are key drivers of plant productivity and ecosystem processes, investigations of these microorganisms in temperate agroforestry systems are scarce, leaving our understanding of agricultural systems under agroforestry practice incomplete. Here, we assessed the composition and diversity of the soil fungal community as well as the frequency (relative abundance) of fungal groups in three paired temperate poplar-based alley cropping (agroforestry) and monoculture cropland systems by amplicon sequencing. Analysis of microbiomes using relative abundances of species or sequence variants obtained from amplicon sequencing ignores microbial population size, which results in several problems. For example, species stimulated by environmental parameters may appear unaffected or suppressed in amplicon counts. Therefore, we determined absolute abundances of selected fungal groups as well as total fungal population size by real-time polymerase chain reaction (PCR). Tree rows strongly affected the community composition and increased the population size and species richness of soil fungi. Furthermore, ectomycorrhiza were strongly promoted by the tree rows. We speculate that mycorrhiza improved the nutrient acquisition in unfertilized tree rows, thereby contributing to the total productivity of the system. Comparison of relative and absolute abundances revealed dramatic discrepancies, highlighting that amplicon sequencing alone cannot adequately assess population size and dynamics. The results of our study highlight the necessity of combining frequency data based on amplicon sequencing with absolute quantification.

## 1. Introduction

Agroforestry is an alternative agricultural land use that combines crops with trees. Alley cropping is a popular type of agroforestry in which rows of crops are alternated with rows of trees. Modern alley-cropping systems in the temperate zone combine rows of fast-growing trees (e.g., hybrid poplar (*Populus* spp.)) with rows of arable crops. The spatial proximity of trees and crops triggers diverse above- and belowground interactions between the two habitats, resulting from joint use of resources such as light, water and nutrients as well as from direct biotic interactions such as predation and parasitism [1]. The main advantage of agroforestry compared to conventional monoculture croplands is its potential for a complementary use of resources between trees and crops, which eventually results in a greater resource use efficiency of the system [2].

The environmental benefits of temperate agroforestry systems have been extensively reviewed [3,4,5]. For example, the integration of trees in arable land has been shown to reduce nitrate leaching [6,7,8] and enhance soil fertility [9]. Likewise, temperate agroforestry has been shown to increase pollination services [10,11] and conserve arthropod diversity [12]. Apart from environmental benefits, crop yield depression has consistently been reported, with the largest yield losses at the tree/crop interface [13,14,15,16]. In an overall assessment of its economic profitability and environmental benefits, temperate agroforestry is, however, perceived as a sustainable alternative to conventional monoculture cropping [5,17].

It has recently been shown that rows of poplar trees of agroforestry systems increase the bacteria-to-fungi ratio in soil as compared to crop rows of agroforestry systems and monoculture croplands [18,19]. Furthermore, poplar trees promoted the abundance of soil bacteria and fungi, particularly Basidiomycota, as compared to crop rows of agroforestry systems and monoculture croplands [20]. Likewise, Beuschel et al. [18] found greater abundance of saprotrophic and ectomycorrhizal (EM) fungi under the trees as compared to the crop rows of poplar-based alley cropping systems. Greater abundance of arbuscular mycorrhizal (AM) fungi in two Canadian agroforestry systems than in monoculture croplands has been found using phospholipid fatty acid analysis [21]. Another study of a Canadian agroforestry system confirmed this finding by the analysis of soil fungal populations using terminal restriction fragment length polymorphism analysis of amplified fungal 28S rRNA genes from plant roots [22]. In a recent study, amplicon sequencing revealed that walnut (*Juglans nigra*) tree rows of French agroforestry systems stimulated mycorrhiza of wheat roots, although the richness of AM fungal communities was higher in the crop rows than in the tree row and at the tree–crop interface and tree roots were only temporarily colonized by mycorrhiza [23]. We speculate that the high content of toxic quinones in walnut tree roots, which has been known for over a century [24], have been responsible for the unexpected result. AM fungi are currently the most frequently studied soil fungal group in temperate agroforestry systems.

Although soil fungal communities include numerous beneficial plant symbionts, pathogens and their antagonists and are key drivers of ecosystem processes [25], studies investigating soil fungal communities of temperate agroforestry systems with high taxonomical resolution are scarce. This study aimed to investigate the abundance, diversity and composition of soil fungal communities in three poplar-based alley cropping systems (agroforestry cropland systems) in Germany using amplicon sequencing and quantitative real-time polymerase chain reaction (qPCR).

## 2. Materials and Methods

### 2.1. Study Sites and Study Design

**Figure 1:**
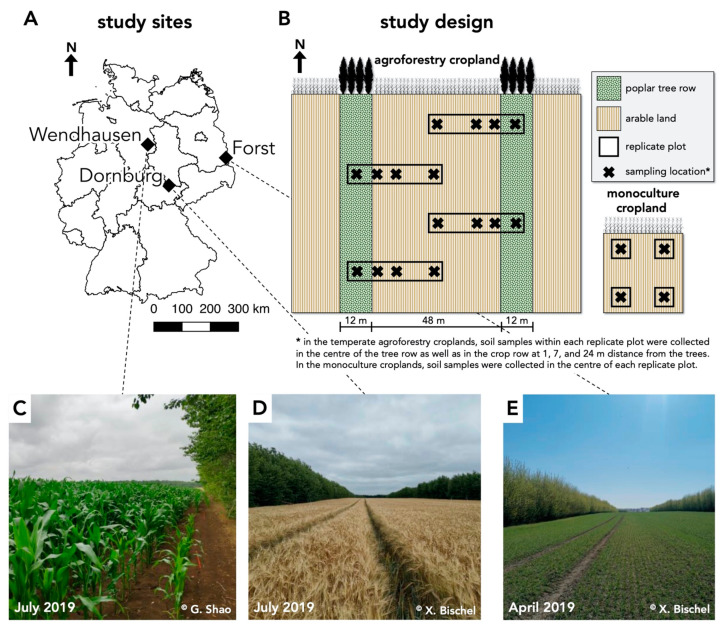
Study sites and study design of paired temperate agroforestry and monoculture croplands. (**A**,**C**–**E**) Study sites on a Calcaric Phaeozem (near Dornburg, Thuringia), Gleyic Cambisol (near Forst, Brandenburg), and Vertic Cambisol soil (near Wendhausen, Lower Saxony) in Germany. Black lines on the map represent borders of federal states. (**B**) Soil samples within each replicate plot of the temperate agroforestry croplands were collected in the centre of the tree row as well as in the crop row at 1, 7, and 24 m distance from the trees in order to cover the spatial heterogeneity of the systems. In the monoculture croplands, soil samples were collected in the centre of each replicate plot.

Our study was conducted at three paired agroforestry and conventional monoculture cropland sites in Germany (Figure 1A). The three sites were located near Dornburg (Thuringia) on a Calcaric Phaeozem soil, near Forst (Brandenburg) on a Gleyic Cambisol soil, and near Wendhausen (Lower Saxony) on a Vertic Cambisol soil (Table S1). The agroforestry systems were poplar-based alley cropping systems that were established between 2007 and 2010. The same poplar clone (clone Max1; *Populus nigra* × *P*. *maximowiczii*) was planted manually from cuttings at every site. The 12 m-wide poplar rows were North–South oriented and alternated with 48 m-wide rows of crops (Figure 1B). The crop rows of each agroforestry system were managed identically as the corresponding monoculture cropland (identical crop rotation, soil management, and fertilization) (Table S1). Tillage and fertilization were conducted in the monoculture croplands and in the crop rows of the agroforestry systems in agreement with common temperate agroforestry practice [4]. Tree rows were not fertilized or tilled. Fertilizer was applied in spring according to standard practice (Table S1).

At every site, four replicate plots were established both in the agroforestry and the monoculture cropland systems (Figure 1B). In the monoculture cropland systems, soil samples were collected within the centre of the plots. In the agroforestry systems, samples were collected along linear transects orthogonal to the tree rows since spatial variations as a function of the distance to the trees have frequently been reported [9]. The transects spanned from the centres of the tree rows to the centres of the crop rows and soil samples were collected in the centre of the tree rows as well as from the crop rows at 1 m, 7 m, and 24 m distance from the trees (Figure 1B).

### 2.2. Soil Collection, DNA Extraction, and Test for Polymerase Chain Reaction (PCR) Inhibitors

Soil sampling was conducted between mid-July to early August 2019 (Calcaric Phaeozem on 16 July, Gleyic Cambisol on 6 August, and Vertic Cambisol on 15 July). Samples of the top 5 cm depth were collected at each of the 20 sampling locations per site, totalling 60 samples. Representative sampling at each sampling location was achieved by collecting 750 cm^3^ of fresh soil, thorough homogenization of the soil in a sterile plastic bag immediately after collection, and aliquotation of approximately 20 g fresh soil into a sterile 15 mL Falcon tube (SARSTEDT, Nümbrecht, Germany). Falcon tubes containing soil samples were stored at −20 °C in the field, during the transportation to the laboratory as well as in the laboratory. Frozen soil samples were freeze-dried and pulverized employing a swing mill (Retsch MM400, Retsch, Haan, Germany) for 1 min at 25 Hz. Soil DNA was extracted from 50 mg finely ground material using a modified protocol of Brandfass and Karlovsky [26]. Soil was suspended in 1 mL cetyltrimethylammonium bromide (CTAB)-based buffer with 1 µL proteinase K and 2 µL 2-mercaptoethanol. The suspension was incubated at 42 °C for 10 min and subsequently at 65 °C for 10 min with frequent inversion. Following the incubation, 800 µL phenol were added and the mixture was shaken and centrifuged. The supernatant was extracted with chloroform/isoamyl alcohol twice and DNA was precipitated using PEG/NaCl. The precipitated DNA was pelleted by centrifugation, washed with EtOH twice and dried using a vacuum centrifuge at 30 °C. The pellets were re-dissolved in 50 µL Tris-EDTA (TE) buffer (10 mM Tris, 1 mM ethylenediaminetetraacetic acid (EDTA), adjusted to pH 8.0 with HCl) and visualized in 0.8% (*w/v*) agarose gels (in Tris-acetate-EDTA (TAE) buffer (40 mM Tris, 20 mM sodium acetate, 1 mM Na_2_EDTA, adjusted to pH 7.6)) stained with ethidium bromide. The extracts were stored at −20 °C. The effect of co-extracted PCR-inhibiting substances on the amplification of DNA was determined using a qPCR inhibition test [27]. The extracts were diluted 1:50 (*v/v*) in 0.5 × TE prior to amplification to overcome PCR inhibition.

### 2.3. Library Preparation and Amplicon Sequencing

Library preparations were carried out in a peqSTAR 96 thermocycler (PEQLAB, Erlangen, Germany) in 25 µL reaction volumes consisting of 18.75 µL mastermix and 6.25 µL of template DNA diluted 1:50 (*v/v*) in 0.5 × TE or 0.5 × TE for the negative control. The mixture contained ddH_2_O, buffer (10 mM Tris-HCl, 50 mM KCl, 1.5 mM MgCl_2_, pH 8.3 at 25 °C), 0.5 mM additional MgCl_2_ to achieve 2 mM Mg^2+^, 200 µM of each deoxynucleoside triphosphate (Bioline, Luckenwalde, Germany), 0.4 µM of each primer (fITS7 [28]/ITS4 [29]), 1 mg mL^−1^ bovine serum albumin, and 0.03 u µL^−1^ Hot Start *Taq* DNA Polymerase (New England Biolabs, Beverly, Massachusetts, USA). The primers were dual-indexed and included 0–3 frameshifting bases (N’s) to improve base-calling during Illumina sequencing followed by an 8-bp barcode sequence at the 5′-end of each primer to allow multiplexing. Thermocycling conditions were as follows: initial denaturation (95 °C for 2 min), 3 touch-up cycles of denaturation (95 °C for 20 s), annealing (50 °C for 30 s), and elongation (68 °C for 30 s), 30 cycles of denaturation (95 °C for 20 s), annealing (58 °C for 30 s), and elongation (68 °C for 30 s), and final elongation (68 °C for 5 min). Aliquots of 4 µL PCR product were visualized on 1.7% (*w/v*) agarose gels (in TAE buffer) stained with ethidium bromide. Gel electrophoresis was carried out at 4.6 V cm^−2^ for 60 min. Libraries were normalized by densitometry using ImageJ version 1.52q [30] as described previously [31]. Briefly, different volumes (1, 2, 3, 4, and 5 µL) of an amplified library on 1.7% (*w/v*) agarose gels served as a standard for the densitometric quantification of library DNA. A linear regression was fitted to obtain a calibration curve and the relative library yield of the samples was determined. The libraries were pooled according to their relative library yield sent to LGC Genomics (Berlin, Germany) for adapter ligation using a commercial kit (Ovation^®^ Rapid DR Multiplex System 1-96 (NuGEN, San Carlos, CA, USA)). The libraries were sequenced using the Illumina MiSeq Reagent Kit v. 3 (2 × 300 bp) (Illumina, San Diego, CA, USA) at the facilities of LGC Genomics, Berlin, Germany. Amplicon sequencing data have been deposited at NCBI’s Short Read Archive (BioProject PRJNA667608).

### 2.4. Processing of Amplicon Sequencing Data

**Figure 2:**
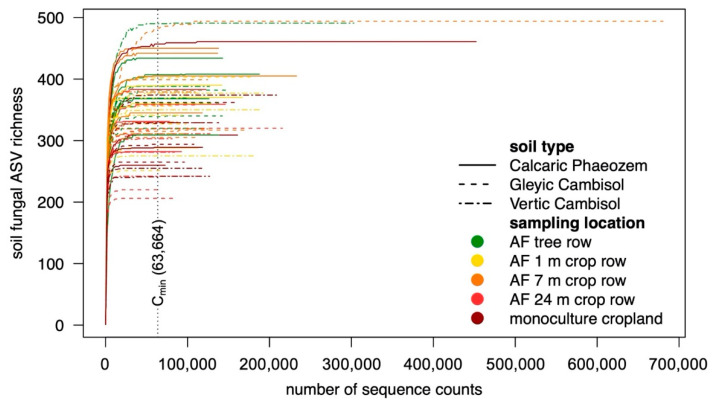
SRS curves and number of sequence counts used for normalization (C_min_) using SRS. Step size = 2000 counts. AF = agroforestry system, ASV = amplicon sequence variant, SRS = scaling with ranked subsampling.

Raw paired-end data were demultiplexed using Illumina’s bcl2fast v. 2.17.1.14 (Illumina, San Diego, CA, USA) and sorted by their barcodes (allowing 1 mismatch per barcode; missing, one-sided or conflicting barcode pairs were discarded). Illumina sequencing adapters were clipped, reads shorter than 100 bp were discarded and primers were clipped (allowing 3 mismatches per primer). Reads were imported in QIIME 2 v. 2019.10 [32]. The read quality was evaluated with ‘q2-demux’ plugin and the reads were truncated to 200 bp, quality filtered (allowing 1 expected error per read), forward and reverse read were merged and chimeras and singletons were removed using DADA2 [33]. The reads were clustered into exact amplicon sequence variants (ASVs). The ASVs obtained were taxonomically classified against the UNITE database v. 8.2 QIIME developer release [34] using a scikit-learn naive Bayes machine-learning classifier (‘q2-fit-classifier-naive-bayes’ and ‘q2- classify-sklearn’ plugin) applying the ‘precision’ configuration to maximize classification precision as proposed by Bokulich et al. [35]. After non-fungal reads were removed, the ASV table contained 8,764,081 counts. Scaling with ranked subsampling (SRS) curves were drawn (Figure 2) and the count data were normalized to 63,664 counts per sample using SRS [36] employing the ‘SRS’-function in the ‘SRS’ R package v. 0.1.1 in the R environment version 3.6.1 [37]. The normalized dataset contained 4969 fungal ASVs.

### 2.5. Quantification of Soil Fungi

The abundance of all soil fungi as well as Asco- and Basidiomycota was determined using qPCR as described previously [20]. We further quantified 10 selected fungal genera/species: *Alternaria* spp., *Cladosporium* spp., *Fusarium culmorum*, *F. graminearum*, *F. tricinctum*, *F. oxysporum*, *Leptosphaeria biglobosa*, *L. maculans*, *Trichoderma* spp., and *Verticillium longisporum*. qPCR reactions were performed in 4 µL reaction volume in 384-well microplates using a CFX384 Thermocycler (Bio-Rad, Rüdigheim, Germany). The reaction volume contained 3 µL mastermix and 1 µL of a 1:50 dilution in 0.5 × TE buffer of the DNA extracts or 0.5 × TE buffer for negative controls. The qPCR assays were performed using either a conventional or hot start polymerase with ThermoPol^®^ Reaction Buffer (20 mM Tris-HCl, 10 mM (NH_4_)_2_SO_4_, 10 mM KCl, 2 mM MgSO_4_, 0.1% Triton^®^ X-100; pH 8.8) or Standard *Taq* Buffer (10 mM Tris-HCl, 50 mM KCl, 1.5 mM MgCl_2_; pH 8.3), varying final concentrations of MgCl_2_, varying concentrations of an equimolar mixture of all four deoxyribonucleoside triphosphates (Bioline, Luckenwalde, Germany), varying concentrations of primers, bovine serum albumin (1 µg/µL reaction volume), 0.1 × SYBR Green I solution (Invitrogen, Karlsruhe, Germany) or 0.25 × EvaGreen solution (Jena Bioscience, Jena, Germany), and 0.03 U *Taq* or Hot Start *Taq* DNA Polymerase/µl reaction volume (New England Biolabs, Beverly, MA, USA). Standards were obtained either from purified PCR products (all soil fungi, Ascomycota, Basidiomycota, *Alternaria* spp., *F. oxysporum*, and *Trichoderma* spp.) or genomic DNA extracted from pure cultures (*Cladosporium* spp., *F. culmorum*, *F. graminearum*, *F. tricinctum*, *L. biglobosa*, *L. maculans*, and *V. longisporum*). For details regarding the composition of the mastermix, primers, and thermocycling conditions see Tables S2–S4.

### 2.6. Statistical Analysis

Shannon diversity index (*H’*), species richness, and Pielou’s evenness index (*J’*) were calculated in the ‘vegan’ R package v. 2.5–6 [38]. The effect of sampling location (tree row, 1 m, 7 m, and 24 m distance from the tree row within the crop row and monoculture cropland) on the diversity, richness, evenness as well as the relative and absolute abundance of selected fungal groups was tested in linear mixed effect (LME) models, with sampling location as fixed effect and site as random effect. Differences among sampling locations were assessed using the ‘glht’-function (general linear hypotheses) in the ‘multcomp’ R package v. 1.4–12 [39] with *p*-value correction for multiple comparison using the Benjamini–Hochberg method [40]. Relationships between distance from the tree row and alpha diversity indices (diversity, richness and evenness) as well as distance and relative and absolute abundances were tested using the Spearman rank correlation. Alpha diversity measures and relative and absolute abundances were normalized by diving the data by the mean per site; distances from the tree rows were ranked (1^st^ rank: tree row, 2^nd^ rank: 1 m crop row, 3^rd^ rank: 7 m crop row, 4^th^ rank: 24 m crop row, 5^th^ rank: monoculture cropland) prior to correlation analysis.

ASV count data were square root transformed and the Bray–Curtis index of dissimilarity was calculated pairwise employing the ‘vegdist’-function in the ‘vegan’ R package v. 2.5–6 [38]. Two different rank-based ordination techniques were applied to visualize patterns of community composition based on Bray–Curtis dissimilarities: i) non-metric multidimensional scaling (NMDS) (‘metaMDS’-function in the ‘vegan’ R package v. 2.5–6 [38]) and ii) canonical analysis of principal coordinates (CAP) [41] (‘CAPdiscrim’-function in the ‘BiodiversityR’ R package v. 2.11–3 [42]). Permutational multivariate analysis of variance (PERMANOVA) was performed with 999 permutations (‘adonis2′-function in the ‘vegan’ R package v. 2.5–6 [38]) to test the effect of soil type, sampling location, and soil type × sampling location on the composition of the soil fungal community. Additionally, pairwise PERMANOVA (‘pairwise.perm.manova’-function in the ‘RVAideMemoire’ R package v. 0.9–75 [43]) with p-value correction for multiple comparison using the Benjamini–Hochberg method [40] was performed to determine differences among sampling locations on the community composition.

We inspected the frequency (relative abundance) of all classified soil fungal genera. The effect of sampling locations within one soil type on the relative abundance of a genus was determined using one-way analysis of variance (ANOVA) with Tukey’s honestly significant difference (HSD) test or the Kruskal–Wallis test with multiple comparison extension. Depending on data properties, data were partially log or square root transformed prior to ANOVA. The relative abundances of selected genera were Z-score normalized for visualization purposes. Z-scores for the relative abundance of each genus were calculated by subtracting the mean relative abundance of the genus from the relative abundance of this genus in each sample and dividing it by the standard deviation. Statistical analysis was performed in the R environment v. 3.6.1 [37].

## 3. Results

### 3.1. Community Composition and Diversity of Soil Fungi

**Figure 3:**
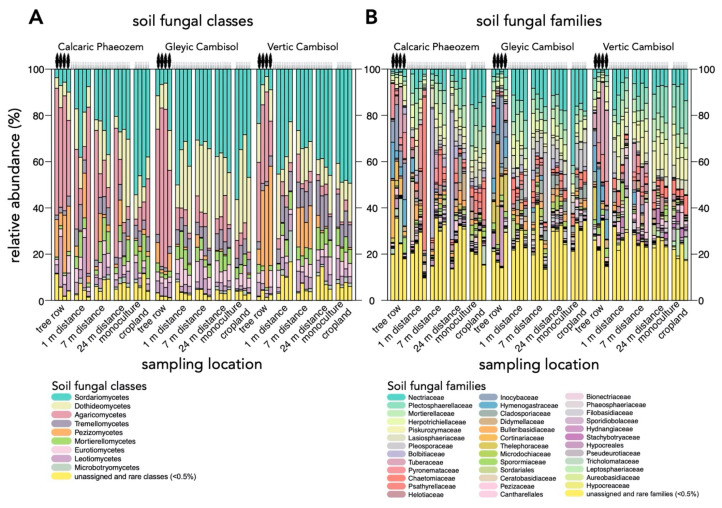
Relative abundance of dominant (>0.5%) soil fungal (**A**) classes and (**B**) families in in paired temperate agroforestry and monoculture cropland systems. Stacked bars represent individual samples. Soil samples within each replicate plot of the temperate agroforestry croplands were collected in the centre of the tree row as well as in the crop row at 1, 7, and 24 m distance from the trees in order to cover the spatial heterogeneity of the systems. In the monoculture croplands, soil samples were collected in the centre of each replicate plot.

The 4969 fungal ASVs included species assigned to 14 fungal phyla of which Ascomycota (66.1 ± 15.3%), Basidiomycota (25.9 ± 16.9%), and Mortierellomycota (5.5 ± 2.9%) were most abundant. The three most abundant classes were Sordariomycetes (Ascomycota; 30.7 ± 14.4%), Dothideomycetes (Ascomycota; 17.0 ± 9.6%), and Agaricomycetes (Basidiomycota; 17.0 ±19.8%) (Figure 3A). The most abundant families were Nectriaceae (Ascomycota; 9.0 ± 7.0%), Plectosphaerellaceae (Ascomycota; 6.8 ± 4.9%), and Mortierellaceae (Mortierellomycota; 5.5 ± 2.9%) (Figure 3B). Among 488 genera, *Mortierella* (Mortierellomycota; 5.2 ± 2.8%), *Exophiala* (Ascomycota; 4.5 ± 2.2%), and *Solicoccozyma* (Basidiomycota; 3.9 ± 4.0%) were the most abundant. The most abundant species were *Mortierella minutissima* (Mortierellomycota; 1.8 ± 1.5%) followed by the three ectomycorrhizal fungi *Inocybe curvipes* (Basidiomycota; 1.7 ± 4.7%), *Geopora cervina* (Basidiomycota; 1.6 ± 3.5%), and *Cortinarius diasemospermus* spp. (Basidiomycota; 1.0 ± 4.2%).

**Figure 4:**
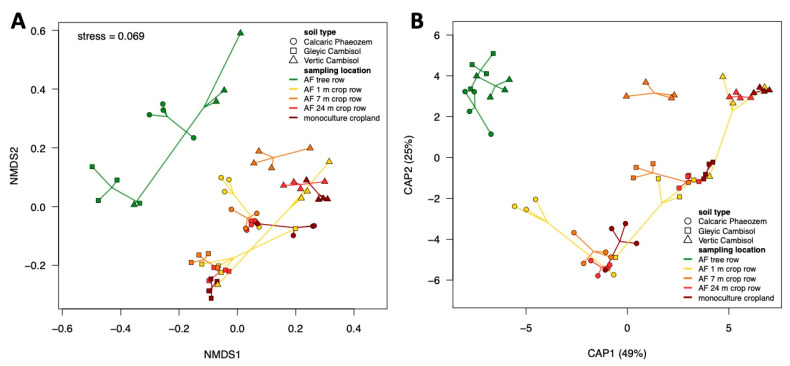
Beta diversity of soil fungal communities in paired temperate agroforestry and monoculture cropland systems. (**A**) Non-metric multidimensional scaling (NMDS) and (**B**) canonical analysis of principal coordinates (CAP) for pairwise Bray–Curtis dissimilarities; samples from each location are connected with their centroids. In all panels, circles, squares and triangles represent individual samples (*n* = 4) from Calcaric Phaeozem, Gleyic Cambisol and Vertic Cambisol, respectively. Soil samples within each replicate plot of the temperate agroforestry croplands were collected in the centre of the tree row as well as in the crop row at 1, 7, and 24 m distance from the trees in order to cover the spatial heterogeneity of the systems. In the monoculture croplands, soil samples were collected in the centre of each replicate plot. AF = agroforestry system.

The composition of the soil fungal community was mainly affected by the site (F = 9.1; *p* = 0.001) followed by the sampling location across sites (F = 3.6; *p* = 0.001). The visualization of the patterns of community composition using both NMDS and CAP, reflected these results (Figure 4A,B). Furthermore, both ordination techniques indicated that the differences between the tree rows and the arable land (crop rows of the agroforestry systems and monoculture croplands) likely accounted for the differences in community composition among sampling locations (Figure 4A,B). Pairwise PERMANOVA with sampling locations across sites as grouping factor confirmed this assumption since the tree row was different from all other sampling locations (F = 3.9–5.5; *p* = 0.0025) but the sampling locations within the arable land did not differ from each other (F = 0.7–1.7; *p* ≥ 0.1).

**Figure 5:**
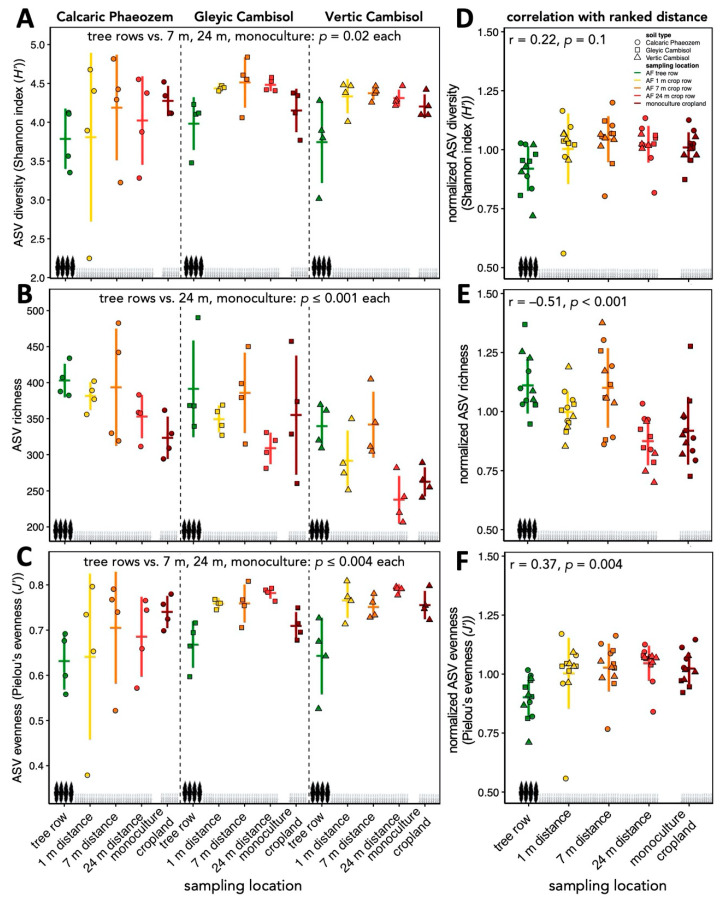
Alpha diversity of soil fungal communities and their correlation with distance from the tree rows. (**A**–**C**) Alpha diversity measures for fungal ASVs. Differences between sampling locations (tree row, 1 m, 7 m, and 24 m distance from the tree row within the crop row and monoculture cropland) were tested using linear mixed effect models with sampling location as fixed effect and site as random effect. Only differences between tree rows and arable land (1 m, 7 m, and 24 m distance from the tree row within the crop row and monoculture cropland) are reported in the panels. (**D**–**F**) Spearman rank correlations of alpha diversity measures with distances from the tree rows. Alpha diversity measures were normalized by the mean per site; distances from the tree rows were ranked (1^st^ rank: tree row, 2^nd^ rank: 1 m crop row, 3^rd^ rank: 7 m crop row, 4^th^ rank: 24 m crop row, 5^th^ rank: monoculture cropland) prior to correlation analysis. In all panels, circles, squares and triangles represent individual samples (*n* = 4) from Calcaric Phaeozem, Gleyic Cambisol and Vertic Cambisol soil, respectively. Horizontal and vertical bars represent the mean and standard deviation, respectively. ASV = amplicon sequence variant.

The establishment of the agroforestry systems affected the alpha diversity of soil fungal ASVs. The tree rows showed lower Shannon diversity and evenness than the crop rows at 7 and 24 m distance from the trees and monoculture croplands (*p* ≤ 0.02) (Figure 5A,C). Richness of fungal ASVs was greater in the tree rows as compared to the centre of the crop rows and the monoculture croplands (*p* ≤ 0.001) (Figure 5B) and showed a negative correlation with distance from the tree rows (r = −0.51, *p* < 0.001) (Figure 5E). Evenness was positively correlated with distance from the tree rows (r = 0.37, *p* = 0.004) (Figure 5F).

### 3.2. Comparison of Absolute and Relative Abundance of Selected Taxonomic Groups of Soil Fungi

**Figure 6:**
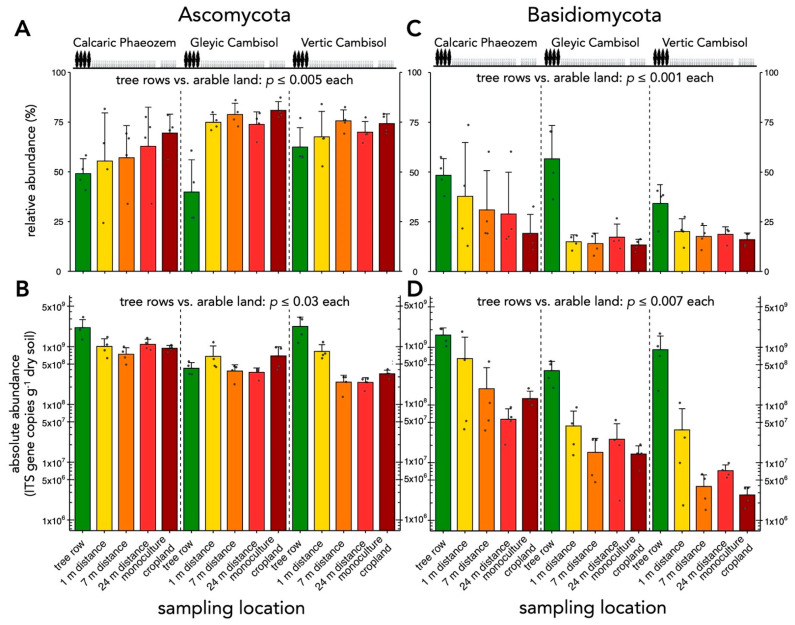
Abundance of Asco- and Basidiomycota. (**A**) Relative and (**B**) absolute abundance of Ascomycota. (**C**) Relative and (**D**) absolute abundance of Basidiomycota. Relative abundances were calculated from ASV counts obtained by amplicon sequencing. The absolute abundance, expressed as gene copy number, was determined by real-time polymerase chain reaction (PCR). Differences between sampling locations (tree row, 1 m, 7 m, and 24 m distance from the tree row within the crop row and monoculture cropland) were tested using linear mixed effect models with sampling location as fixed effect and site as random effect. Only differences between tree rows and arable land (1 m, 7 m, and 24 m distance from the tree row within the crop row and monoculture cropland) are reported in the panels. Bars represent the mean and error bars show standard deviation (*n* = 4). Grey dots represent individual data points.

Soil fungi were more abundant in the tree rows than in the arable land (crop rows of agroforestry and conventional monoculture croplands) (*p* ≤ 0.03) (Figure S1A) and their abundance decreased with increasing distance from the trees (r = −0.42, *p* = 0.0001) (Figure S1B). The relative as well as absolute abundance of Basidiomycota was greater in the tree rows than in the arable land (*p* ≤ 0.007) (Figure 6C,D). Both absolute and relative abundance declined with increasing distance from the trees (r = −0.53–−0.56, *p* < 0.0001) (Figure S2C,D). While the absolute abundance of Ascomycota increased under the trees as compared to the arable land (*p* ≤ 0.03) (Figure 6B), their relative abundance was lower in the tree rows than in the arable land (*p* ≤ 0.005) (Figure 6A). Similarly, increasing distance from the trees was negatively associated with absolute abundance (r = −0.37, *p* = 0.004) (Figure S2B) and positively associated with relative abundance of Ascomycota (r = 0.48, *p* = 0.0001) (Figure S2A).

**Figure 7:**
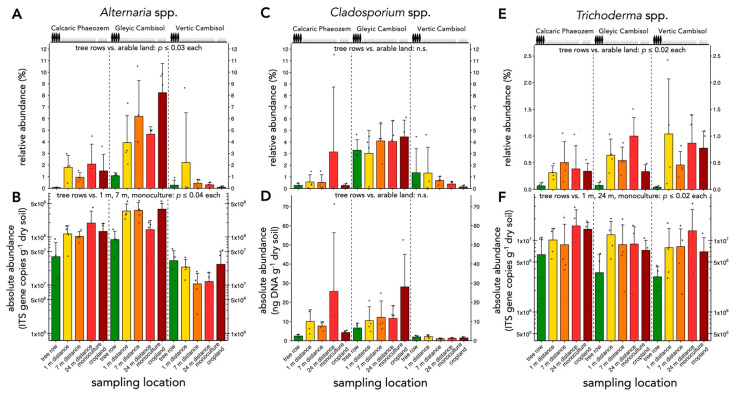
Abundance of *Alternaria*, *Cladosporium* and *Trichoderma* spp. (**A**) Relative and (**B**) absolute abundance of *Alternaria* spp. (**C**) Relative and (**D**) absolute abundance of *Cladosporium* spp. (**E**) Relative and (**F**) absolute abundance of *Trichoderma* spp. Relative abundances were calculated from ASV counts obtained by amplicon sequencing. The absolute abundance, expressed as gene copy number, was determined by real-time PCR. Differences between sampling locations (tree row, 1 m, 7 m, and 24 m distance from the tree row within the crop row and monoculture cropland) were tested using linear mixed effect models with sampling location as fixed effect and site as random effect. Only differences between tree rows and arable land (1 m, 7 m, and 24 m distance from the tree row within the crop row and monoculture cropland) are reported in the panels. Bars represent the mean and error bars show standard deviation (*n* = 4). Grey dots represent individual data points. ASV = amplicon sequence variant; n.s. = no statically significant differences.

**Figure 8:**
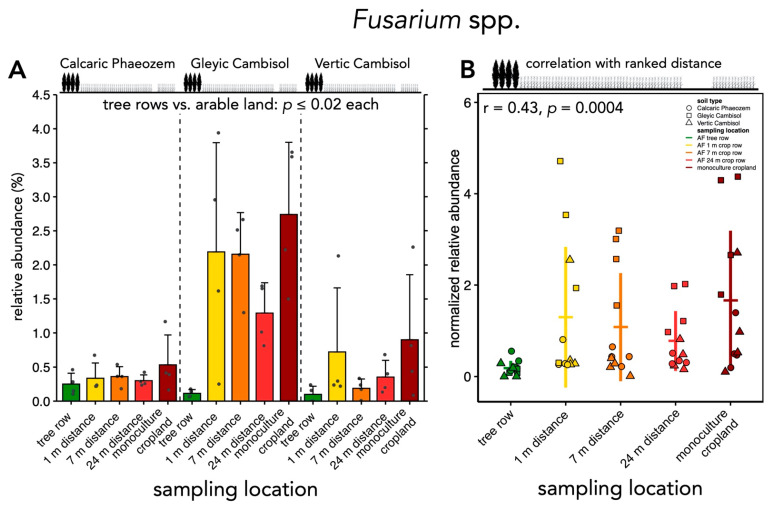
Relative abundance of *Fusarium* spp. and correlation with distance from the tree rows. (**A**) Relative abundances of *Fusarium* spp. were calculated from ASV counts obtained by amplicon sequencing. The mean gene copy number with standard deviation (*n* = 4) are shown. Grey dots represent individual data points. Differences between sampling locations (tree row, 1 m, 7 m, and 24 m distance from the tree row within the crop row and monoculture cropland) were tested using linear mixed effect models with sampling location as fixed effect and site as random effect. Only differences between tree rows and arable land (1 m, 7 m, and 24 m distance from the tree row within the crop row and monoculture cropland) are reported in the panel. (**B**) Spearman rank correlation of relative abundance of *Fusarium* spp. with distance from the tree rows. Absolute fungal 18S rRNA gene abundances were normalized by the mean per site; distances from the tree rows were ranked (1^st^ rank: tree row, 2^nd^ rank: 1 m crop row, 3^rd^ rank: 7 m crop row, 4^th^ rank: 24 m crop row, 5^th^ rank: monoculture cropland) prior to correlation analysis. In all panels, circles, squares and triangles represent individual samples (*n* = 4) from Calcaric Phaeozem, Gleyic Cambisol and Vertic Cambisol soil, respectively. Horizontal and vertical bars represent the mean and standard deviation, respectively.

Affiliates of the genus *Alternaria* showed greater relative abundance in the arable land than in the tree rows (*p* ≤ 0.03) (Figure 7A). Similarly, absolute abundance of *Alternaria* spp. was lower in the tree rows than at 1 and 7 m distance within the crop rows and monoculture systems (*p* ≤ 0.04) (Figure 7B). The establishment of the poplar tree rows did not affect the relative and absolute abundance of *Cladosporium* spp. (Figure 7C,D). The relative abundance of *Trichoderma* spp. was lower in the tree rows than in the arable land (*p* ≤ 0.02) (Figure 7E). Similarly, absolute abundance was greater in the tree rows than in the arable land, except for the centre of the crop rows (*p* ≤ 0.04) (Figure 7F). Both relative and absolute abundance of *Trichoderma* affiliates were positively associated with increasing distance from the tree rows (r = 0.31–0.47, *p* ≤ 0.02) (Figure S3E,F). The relative abundance of *Fusarium* spp. was lower in the tree rows than the arable land (*p* ≤ 0.02) (Figure 8A) and was positively correlated with the distance from the tree rows (r = 0.43, *p* = 0.0004) (Figure 8B). We detected *V. longisporum* in all samples but rarely above the limit of quantification. While we did not detect *F. graminearum* and *L. maculans*, we sporadically detected *F. culmorum* (in 4 samples), *F. tricinctum* (in 30 samples), *F. oxysporum* (in 26 samples), and *L. biglobosa* (in 3 samples). These results were not analyzed statistically.

### 3.3. Relative Abundances of Selected Soil Fungal Genera under Agroforestry and Monoculture Cropland

**Figure 9:**
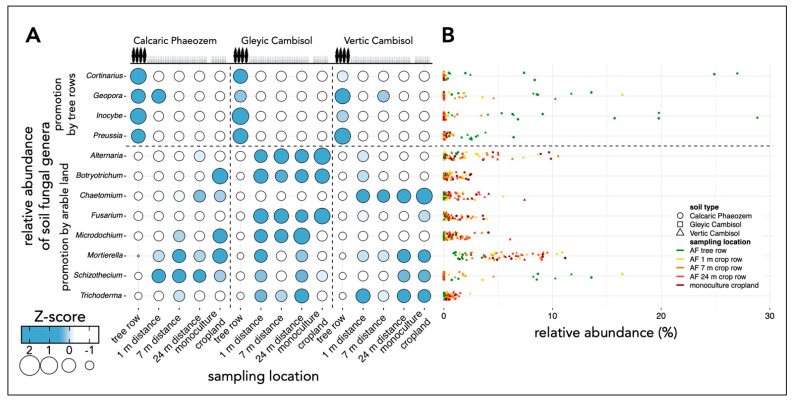
Relative abundance of selected soil fungal genera. (**A**) Mean Z-score normalized relative abundance of selected soil fungal genera (*n* = 4). (**B**) Relative abundance of selected soil fungal genera. Relative abundances were calculated from ASV counts obtained by amplicon sequencing. Soil samples within each replicate plot of the temperate agroforestry croplands were collected in the centre of the tree row as well as in the crop row at 1, 7, and 24 m distance from the trees in order to cover the spatial heterogeneity of the systems. In the monoculture croplands, soil samples were collected in the centre of each replicate plot.

The tree rows of the agroforestry systems on the Calcaric Phaeozem and Vertic Cambisol soil promoted the relative abundance of *Cortinarius* spp. (Basidiomycota) compared to the arable land (*p* ≤ 0.006) (Figure 9). Likewise, species assigned to *Inocybe*, another genus of the phylum Basidiomycota, showed greater relative abundance under the trees than in the arable land on the Calcaric Phaeozem and Gleyic Cambisol soil (*p* < 0.001). On the Vertic Cambisol soil, the relative abundance of *Inocybe* spp. was greater in the tree row than at 1 and 24 m distance from the trees within the crop row and the monoculture cropland (*p* ≤ 0.01). The integration of poplar rows on the Gleyic and Vertic Cambisol soil increased the relative abundance of members of the genus *Preussia* (Ascomycota) as compared to the arable (*p* < 0.001).

Several fungal genera showed lower relative abundance in the tree rows compared to the arable land (Figure 9). For example, on all three soil types, *Microdochium* affiliates were more abundant in the monoculture cropland than under the trees (*p* ≤ 0.048). Additionally, *Microdochium* spp. on the Gleyic Cambisol soil were more abundant in the crop row than in the tree row (*p* < 0.001). The most abundant genus, *Mortierella*, showed greater relative abundance in the arable land than in the tree row on the Vertic Cambisol soil (*p* ≤ 0.048).

## 4. Discussion

Our results agree with previous findings of greater abundance of soil fungi in tree rows of temperate agroforestry systems compared to crop rows and monoculture cropland systems (Figure S1) [18,20,44]. We assume that fungal abundance in the tree rows was favored due to the absence of tillage [45]. Additionally, accumulation of tree litter (leaves, branches, and roots) likely favored saprotrophic fungal decomposers. The strong increase in the absolute abundance of Basidiomycota in the tree rows compared to the arable land (crop rows of the agroforestry systems and conventional monoculture croplands) (Figure 6D) supports this assumption as most wood-decaying and litter-decomposing fungi are Basidiomycota [46].

Soil fungal ASV richness and evenness (Figure 5E,F) and relative and absolute abundances of several fungal groups (Figure 8, Figures S1–S3) were related to the distance from the tree row. Dependence on the distance to tree rows has been described for several parameters in temperate agroforestry systems, including yield (e.g., [15]), soil fertility (e.g., [9]), tree litterfall (e.g., [47]), and soil microbial biomass (e.g., [44]). Tree litterfall decreases exponentially with increasing distance from the trees [47] (Figure S4) and likely serves both as fungal inoculum as well as selective growth substrate for litter-decomposing fungi. Therefore, we suggest that tree litterfall may have contributed to the increase of fungal ASV richness and Basidiomycota with proximity to the tree rows (Figure 5E, Figure S2). Furthermore, tree rows preserve soil fungal communities during disturbance due to management practices conducted in crop rows (e.g., tillage or fungicide application) and may provide fungal inoculum for crop rows following disturbance.

Microbiome count data obtained by amplicon sequencing reflect ratios among abundances of members of the community (relative abundances or frequencies) but they do not reveal the size of populations (absolute abundances) and their changes [48]. This is an often-ignored limitation of microbiome analysis by amplicon sequencing. The lack of information about population size is of particular concern in studies of the effect of environmental parameters such as soil characteristics on microbial communities. For instance, species stimulated by an environmental factor may appear unaffected or even suppressed in amplicon counts. This may happen when other species were stimulated to the same or a higher degree, but also when other species were stimulated to a lower degree yet their population size was larger. Therefore, even qualitative trends such as stimulation versus suppression of a microbial groups cannot be inferred from amplicon sequencing alone. Recently, the problem has been recognized and sequencing of bacterial 16S rRNA amplicons was combined with qPCR [48,49], digital PCR [50] or flow cytometry [51,52], which provided estimates of absolute abundances of bacterial taxa. In line with this reasoning, we supported our microbiome analysis by amplicon sequencing with qPCR assays for selected fungal groups as well as for all fungi.

The comparison of relative with absolute abundances of soil fungal groups revealed similar patterns for Basidiomycota, *Alternaria*, *Cladosporium*, and *Trichoderma* spp., whereas contrasting patterns were found for Ascomycota (Figure 6 and Figure 7). Even for similar patterns of relative and absolute abundances, amplicon count data can skew the magnitude of differences among treatments. For example, on the Vertic Cambisol, the average relative abundance of Basidiomycota in the tree rows was 1.18 times larger than in the monoculture; however, the average absolute abundance of Basidiomycota in the tree rows was 330 times larger than in the monoculture (*cf*. Figure 6C,D). Apart from the inherent limitations of amplicon sequencing, technical issues such as the use of different primers for amplicon sequencing and qPCR [49] and different amplification efficiencies of species may have contributed to this discrepancy. We assume, however, that technical issues alone do not account for the remarkable discrepancies observed and that the very nature of amplicon sequencing data masked the dramatic population dynamics in our study (Figure S1A). Our work highlights the importance of combining frequency data based on amplicon sequencing with absolute quantification.

The abundance of fungal genera harboring phytopathogens of arable crops such as *Alternaria, Fusarium*, and *Microdochium* spp. was reduced by tree rows compared to arable land (Figure 7A,B, Figure 8 and Figure 9), which is likely related to the absence of host plants of these pathogens in the tree rows. Recently, a similar pattern has been found for crop pests which preferentially overwintered in crop rows rather than tree rows of temperate agroforestry systems [53]. In addition to the depletion of phytopathogens in soil, rows of trees dilute the density of arable crops (host dilution effect), which may have contributed to improved suppression of soil-borne diseases in diversified agricultural systems [54]. Therefore, crops cultivated in agroforestry systems may benefit from the presence of trees. We recently tested this hypothesis by investigating the colonization of barley and wheat grain and oilseed rape stems with phytopathogenic fungi in temperate agroforestry and monoculture cropland systems. We demonstrated that agroforestry does not stimulate crop diseases and may even suppress certain phytopathogens such as *V. longisporum* in oilseed rape and *F. tricinctum* in wheat as compared to cropland monoculture [55]. In the present study, we aimed to quantify several phytopathogenic fungi using species-specific qPCR assays but their low biomass in soil mostly prevented amplification. Therefore, the abundance of individual species assigned to genera that harbor phytopathogenic fungi remains unknown. Since phytopathogenic fungi such as *Alternaria* and *Fusarium* spp. have specific hosts that may or may not be present in a given system at a given time, quantification at genus level is not sufficient to evaluate the actual pathogenic potential of the community. Even if the composition of the community has been analyzed, amplicon sequencing of short subunits of taxonomically informative loci rarely allows reliable identification at low taxonomic levels. The lifestyle of taxonomically closely related microorganisms that are indistinguishable using amplicon sequencing can vary, which raises concerns regarding the suitability of amplicon sequencing for the prediction of functional traits of microbial communities.

Both relative and absolute abundances revealed that *Trichoderma* spp. were associated with arable land rather than tree rows (Figure 7E,F). *Trichoderma* spp. are frequently recovered from soil and most species exhibit a mycotrophic lifestyle [56]. Due to their antagonistic effects on other fungi, the genus provides biocontrol agents against plant pathogens such as *Alternaria* spp. [57,58] and *Fusarium* spp. [59,60,61]. Since *Alternaria* spp. and *Fusarium* spp. were more abundant in arable land as compared to tree rows (Figure 7A,B and Figure 8), greater abundance of *Trichoderma* spp. in arable land may reflect the availability of their prey.

Mycorrhizae are of major importance for the production of crops and trees. While arbuscular mycorrhizal (AM) fungi are considered the most important type of mycorrhizae in agriculture, EM fungi are crucial for forestry [62]. Poplar trees form symbiotic root associations with both AM and EM fungi [63]. EM fungi protect plant roots against soil-borne fungal diseases [64] and play a significant role in nutrient uptake, especially regarding nitrogen and phosphorus [65]. There is sufficient evidence that EM fungi are capable of decomposing organic matter for the mobilization of nitrogen [66,67]. The promotion of the ectomycorrhizal genera *Cortinarius*, *Geopora*, and *Inocybe* (Figure 9) by the poplar rows agrees with a recent inventory of EM fungal communities of *Populus simonii* [68]. While the role of *Inocybe* and *Geopora* spp. in nutrient cycling is not well explored, *Cortinarius* spp. are abundant in forest ecosystems [69,70] and considered to play a major role in the decomposition of recalcitrant organic matter for the acquisition of nitrogen [71]. The association of the trees with EM fungi may allow them to benefit from nutrient pools within agroforestry systems that otherwise would be inaccessible to both trees and crops. Therefore, we propose that tree row-associated microorganisms, particularly mycorrhizae, improve nutrient acquisition in the unfertilized tree rows and thereby contribute to the productivity of the tree rows.

## 5. Conclusions

Tree rows in temperate agroforestry cropland systems increased the population size and species richness of soil fungi. Both population size and species richness declined with increasing distance from the tree rows. The dependency on the distance to the trees was likely due to the distribution of tree litter serving both as fungal inoculum and as growth substrate for saprotrophic fungal decomposers. We suggest that tree rows also served as microbial reservoirs, providing fungal inoculum for crop rows after disturbance due to management practices (e.g., tillage or fungicide application). Ectomycorrhizal fungi associated with the tree rows likely improved nutrient acquisition by the trees, thereby contributing to the productivity of tree rows. The dramatic discrepancies found between relative frequencies (obtained by amplicon sequencing) and absolute abundances (genome counts obtained by qPCR) of microbial groups illustrate that population size and dynamics cannot be assessed by amplicon sequencing. These results highlight the importance of combining amplicon sequencing with absolute quantification of selected species as well as total microbial population size.

## Data Availability

Amplicon sequencing data generated during this study have been deposited at NCBI’s Short Read Archive (Bioproject PRJNA667608).

## Supplementary Materials

The following are available online at https://www.mdpi.com/2076-2607/9/3/589/s1, Figure S1: Absolute abundance of soil fungi and correlation with distance from the tree rows, Figure S2: Correlation of abundance of Asco- and Basidiomycota with distance from the tree rows, Figure S3: Correlation of abundance of *Alternaria*, *Cladosporium* and *Trichoderma* spp. with ranked distance from the tree rows, Figure S4: Tree litterfall distribution at the agroforestry cropland system on the Calcaric Phaeozem soil near Dornburg, Table S1: Site characteristics and management at the three study sites of paired temperate agroforestry and monoculture cropland, Table S2: Organisms that served as real-time PCR standards and primer sets used for the real-time PCR assays, Table S3: Mastermix composition used for the real-time PCR assays, Table S4: Thermocycling protocol used for the qPCR assays.
