# Supplementary material for: Relative Abundances of Species or Sequence Variants Can Be Misleading: Soil Fungal Communities as an Example
Source: Microorganisms. 2021 Mar 13;9(3):589. doi: 10.3390/microorganisms9030589 (PMC7999432; doi:10.3390/microorganisms9030589)
Supplement: Supplementary file 1 [file microorganisms-09-00589-s001.pdf]

microorganisms

Supplementary Material

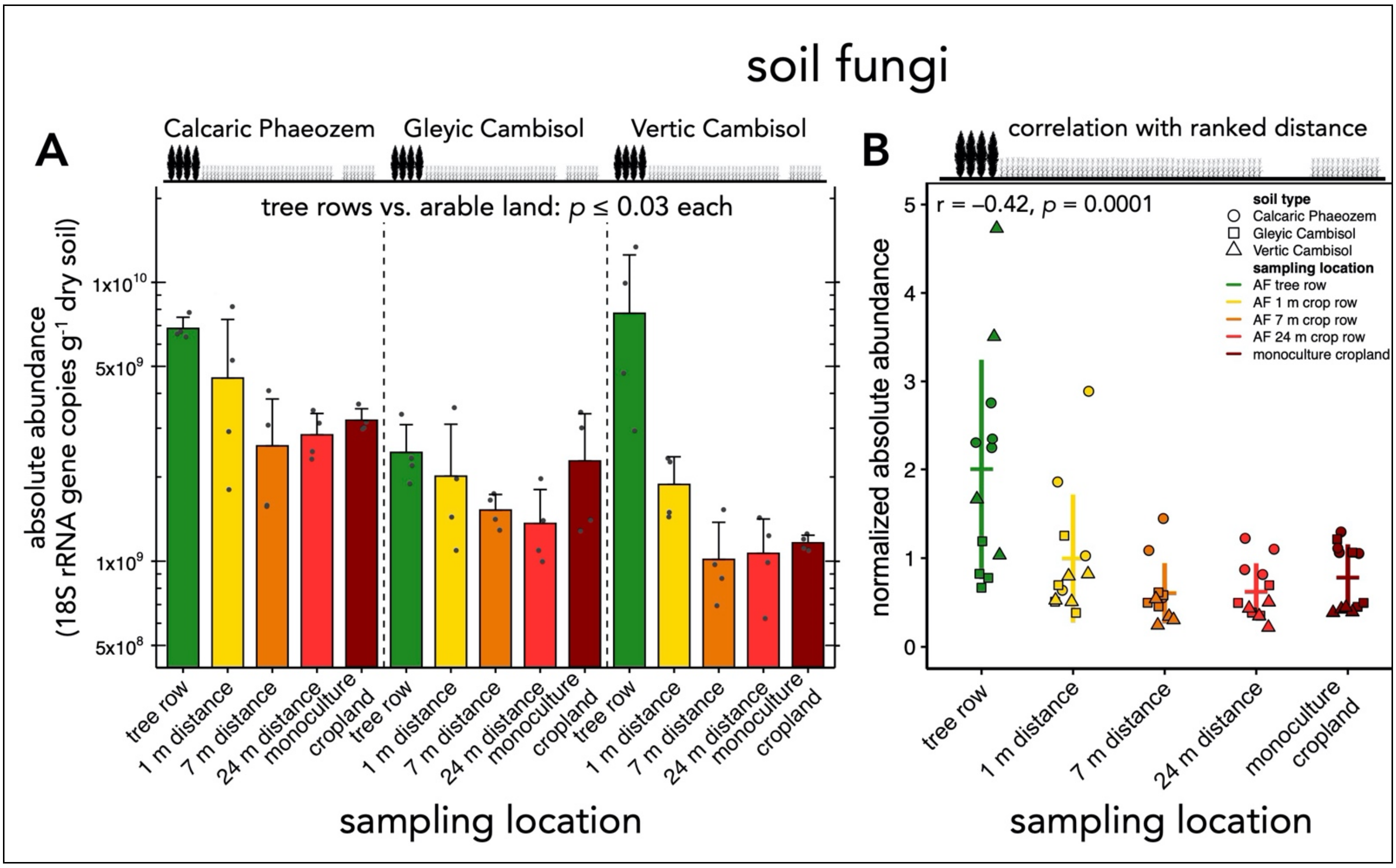

Figure S1. Absolute abundance of soil fungi and correlation with distance from the tree rows. **(A)** Absolute abundances of fungal 18S rRNA genes were obtained using real-time polymerase chain reaction (PCR). The mean gene copy number with standard deviation ($n = 4$) is shown. Grey dots represent individual data points. Differences between sampling locations (tree row, 1 m, 7 m, and 24 m distance from the tree row within the crop row and monoculture cropland) were tested using linear mixed effect models with sampling location as fixed effect and site as random effect. Only differences between tree rows and arable land (1 m, 7 m, and 24 m distance from the tree row within the crop row and monoculture cropland) are reported in the panel. **(B)** Spearman rank correlation of absolute abundance of soil fungi with distance from the tree rows. Absolute fungal 18S rRNA gene abundances were normalized by the mean per site; distances from the tree rows were ranked (1st rank: tree row, 2nd rank: 1 m crop row, 3rd rank: 7 m crop row, 4th rank: 24 m crop row, 5th rank: monoculture cropland) prior to correlation analysis. In all panels, circles, squares and triangles represent individual samples ($n = 4$) from Calcaric Phaeozem, Gleyic Cambisol and Vertic Cambisol soil, respectively. Horizontal and vertical bars represent the mean and standard deviation, respectively.

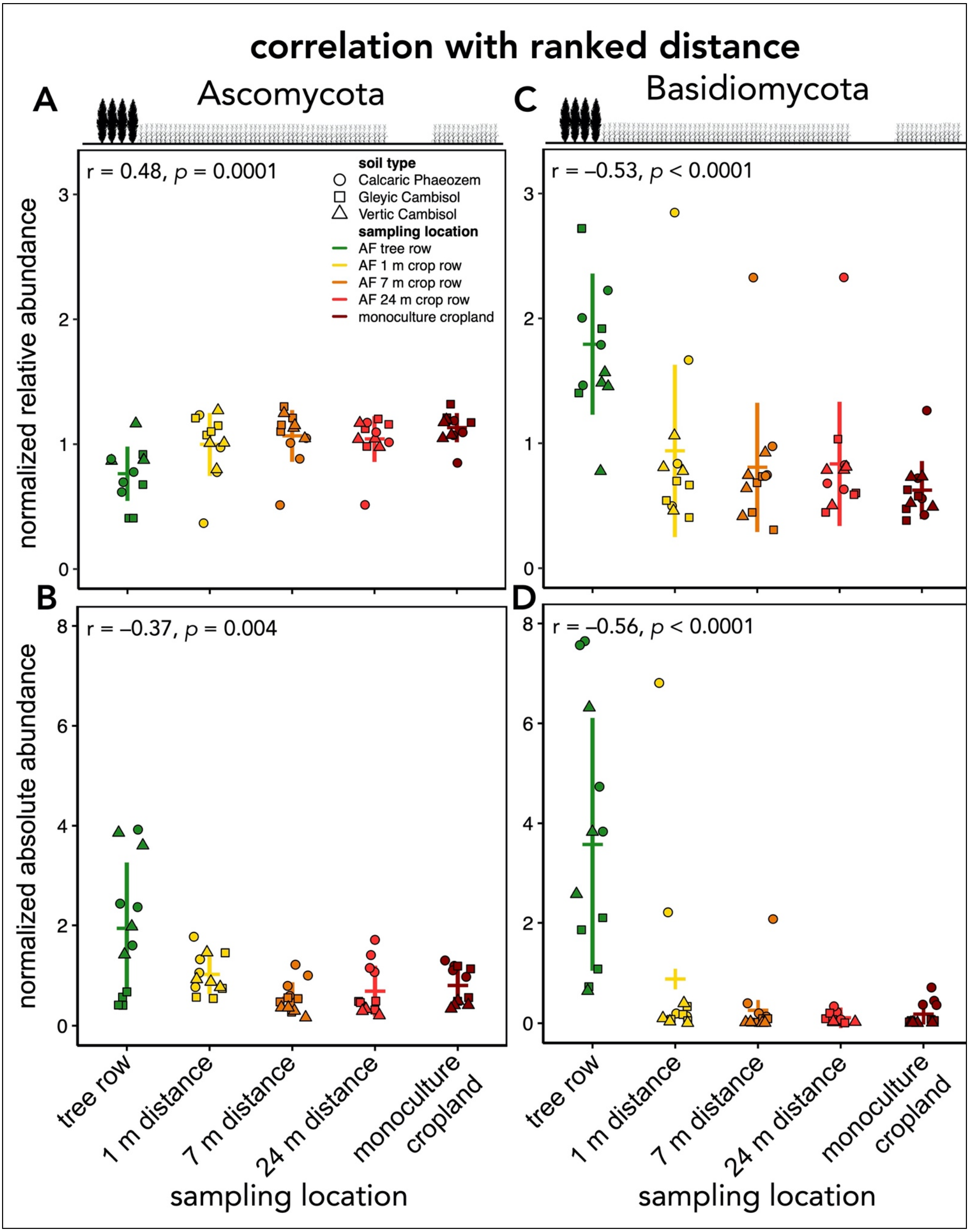

correlation with ranked distance
A
Ascomycota
C
Basidiomycota
r = 0.48, p = 0.0001
soil type
Calcaric Phaeozem
Gleyic Cambisol
Vertic Cambisol
sampling location
AF tree row
AF 1 m crop row
AF 7 m crop row
AF 24 m crop row
monoculture cropland
r = –0.53, p < 0.0001
normalized relative abundance
B
D
r = –0.37, p = 0.004
r = –0.56, p < 0.0001
normalized absolute abundance
tree row
1 m distance
7 m distance
24 m distance
monoculture cropland
sampling location

Figure S2. Correlation of abundance of Asco- and Basidiomycota with distance from the tree rows. Spearman rank correlation of **(A)** relative and **(B)** absolute abundance of Ascomycota with distances from the tree rows, respectively. Spearman rank correlations of **(C)** relative and **(D)** absolute abundance of Basidiomycota with distances from the tree rows, respectively. Relative or absolute abundances were normalized by diving the data by the mean per site; distances from the tree rows were ranked (1st rank: tree row, 2nd rank: 1 m crop row, 3rd rank: 7 m crop row, 4th rank: 24 m crop row, 5th rank: monoculture cropland) prior to correlation analysis. In all panels, circles, squares and triangles represent individual samples (n = 4) from Calcaric Phaeozem, Gleyic Cambisol and Vertic Cambisol soil, respectively. Horizontal and vertical bars represent the mean and standard deviation, respectively.

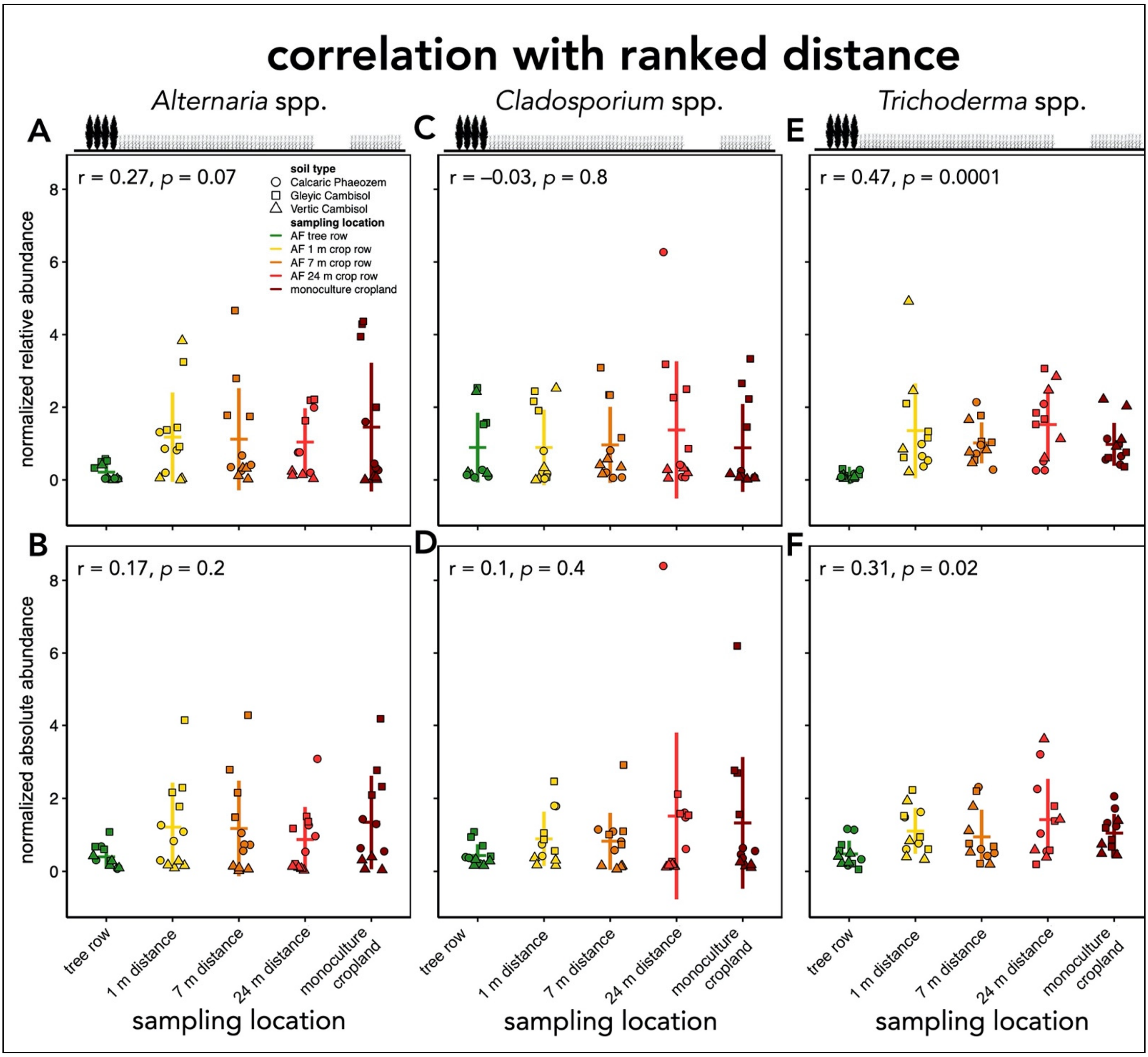

Figure S3. Correlation of abundance of *Alternaria*, *Cladosporium* and *Trichoderma* spp. with ranked distance from the tree rows. Spearman rank correlation of **(A)** relative and **(B)** absolute abundance of *Alternaria* spp. with distances from the tree row, respectively. Spearman rank correlation of **(C)** relative and **(D)** absolute abundance of *Cladosporium* spp. with distances from the tree rows, respectively. Spearman rank correlations of **(E)** relative and **(F)** absolute abundance of *Trichoderma* spp. with distances from the tree rows, respectively. Relative or absolute abundances were normalized by the mean per site; distances from the tree rows were ranked (1st rank: tree row, 2nd

rank: 1 m crop row, 3rd rank: 7 m crop row, 4th rank: 24 m crop row, 5th rank: monoculture cropland) prior to correlation analysis. In all panels, circles, squares and triangles represent individual samples (n = 4) from Calcaric Phaeozem, Gleyic Cambisol and Vertic Cambisol soil, respectively. Horizontal and vertical bars represent the mean and standard deviation, respectively.

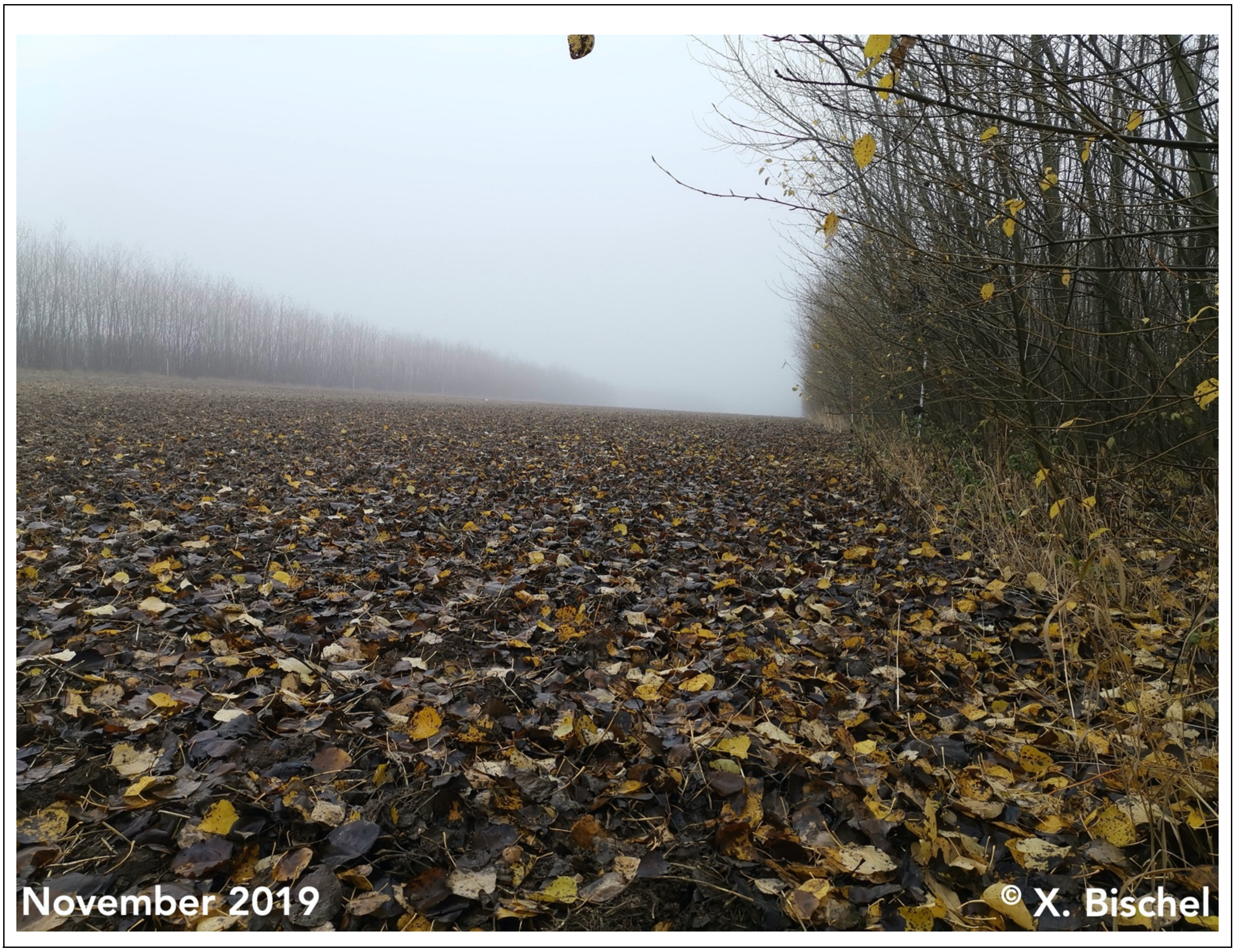

Figure S4. Tree litterfall distribution at the agroforestry cropland system on the Calcaric Phaeozem soil near Dornburg.

Table S1. Site characteristics and management at the three study sites of paired temperate agroforestry and monoculture cropland.

Study Site	Dornburg	Forst	Wendhausen
location	51°00′40″N, 11°38′46″E	51°47′11″N, 14°38′05″E	52°20′00″N, 10°37′55″E
soil type<br>meters above sea level	Calcaric Phaeozem<br>289 m	Gleyic Cambisol<br>67 m	Vertic Cambisol<br>82 m
mean annual air temperature (1981–2010)	9.9 ± 0.1 °C [a]	9.6 ± 0.2 °C [b]	9.6 ± 0.2 °C [c]
mean annual precipitation (1981–2010)	608 ± 21 mm [a]	568 ± 21 mm [b]	637 ± 23 mm [c]
year of agroforestry system establishment	2007	2010	2008
harvest(s) of the aboveground tree biomass of the agroforestry system	January 2015	February 2015, March 2018	January 2014
crop rotation (2016–2017–2018–2019)	summer barley–winter oilseed rape–winter wheat–summer barley	winter wheat–winter barley–maize–summer barley	winter oilseed rape–winter wheat–winter wheat–maize
fertilization rates 2019 (kg N–P–K ha^{-1} yr^{-1})	36–22–31	42–8–27	101–0–0

Mean ± standard error during 1981 to 2010; [a] climate station at Jena (station ID: 2444) of the German Meteorological Service.; climate station at [b] Cottbus (station ID: 880) of the German Meteorological Service.; climate station at [c] Braunschweig (station ID: 662) of the German Meteorological Service.

Table S2. Organisms that served as real-time PCR standards and primer sets used for the real-time PCR assays.

Target Organism(s)	Organisms Used for Standard	Primer Set	Primer Reference
Total fungi	*Verticillium longisporum* VL43 [a]	FR1 / FF390	[1]
Ascomycota	*Fusarium graminearum* IFA66	ITS4Asco / ITS5	[2,3]
Basidiomycota	commercial *Agaricus* spp.	ITS4b / 5.8sr	[4,5]
Alternaria spp.	*Alternaria alternata* [b]	Dir1ITSAlt / Inv1ITSAlt	[6]
Cladosporium spp.	*Cladosporium cladosporioides* IPP170 [a]	Clado-SYBRG-PF / Clado-SYBRG-PR	[7]
Fusarium culmorum	*Fusarium culmorum* DSM 62191	OPT18 F / OPT18 R	[8]
Fusarium graminearum	*Fusarium graminearum* IFA66	Fg16N F / Fg16N R	[9]
Fusarium tricinctum	*Fusarium tricinctum* DSM 23357	Tri1 / Tri2	[10]
Fusarium oxysporum	*Fusarium oxysporum* DSM 62296	PFO2 / PFO3	[11]
Leptosphaeria biglobosa	*Leptosphaeria biglobosa* IPP1560 [a]	LbigF / LmacR	[12]
Leptosphaeria maculans	*Leptosphaeria maculans* T12aD34 [a]	LmacF / LmacR	[12]
Trichoderma spp.	*Trichoderma virens* DSM1963	ITSTrF / ITSTrR	[13]
Verticillium longisporum	*Verticillium longisporum* VL43 [a]	OLG70 / OLG71	[14]

[a] provided by A. von Tiedemann, University of Goettingen, Germany; [b] provided by A.S.H. Abbo, University of Khartoum, Sudan.

[1] Vainio, E.J.; Hantula, J. Direct Analysis of Wood-Inhabiting Fungi Using Denaturing Gradient Gel Electrophoresis of Amplified Ribosomal DNA. Mycological Research 2000, 104, 927–936; [2] White, T.; Bruns, T.; Lee, S.; Taylor, J. Amplification and direct sequencing of fungal ribosomal RNA genes for phylogenetics. In PCR protocols: a guide to methods and applications; Academic Press, New York., 1990; pp. 315–322; [3] Nikolcheva, L.G.; Bärlocher, F. Taxon-Specific Fungal Primers Reveal Unexpectedly High Diversity during Leaf Decomposition in a Stream. Mycol Progress 2004, 3, 41–49, doi:10.1007/s11557-006-0075-y; [4] Vilgalys, R.; Hester, M. Rapid Genetic Identification and Mapping of Enzymatically Amplified Ribosomal DNA from Several Cryptococcus Species. J. Bacteriol. 1990, 172, 4238–4246; [5] Gardes, M.; Bruns, T.D. ITS Primers with Enhanced Specificity for Basidiomycetes--Application to the Identification of Mycorrhizae and Rusts. Mol. Ecol. 1993, 2, 113–118; [6] Pavón, M.; González, I.; Rojas, M.; Pegels, N.; Martín, R.; García, T. PCR Detection of Alternaria Spp. in Processed Foods, Based on the Internal Transcribed Spacer Genetic Marker. J Food Prot 2011, 74, 240–247, doi:10.4315/0362-028X.JFP-10-110; [7] Zeng, Q.-Y.; Westermark, S.-O.; Rasmuson-Lestander, Å.; Wang, X.-R. Detection and Quantification of Cladosporium in Aerosols by Real-Time PCR. J. Environ. Monit. 2006, 8, 153–160, doi:10.1039/B509515H; [8] Schilling, A.G.; Moller, E.M.; Geiger, H.H. Polymerase Chain Reaction-Based Assays for Species-Specific Detection of Fusarium Culmorum, F. Graminearum, and F. Avenaceum. Phytopathology 1996, 86, 515–22; [9] Nicholson, P.; Simpson, D.R.; Weston, G.; Rezanoor, H.N.; Lees, A.K.; Parry, D.W.; Joyce, D. Detection and Quantification of Fusarium Culmorum and Fusarium Graminearum in Cereals Using PCR Assays. Physiological and Molecular Plant Pathology 1998, 53, 17–37, doi:10.1006/pmpp.1998.0170; [10] Kulik, T. Detection of Fusarium Tricinctum from Cereal Grain Using PCR Assay. J. Appl. Genet. 2008, 49, 305–311, doi:10.1007/BF03195628; [11] Edel, V.; Steinberg, C.; Gautheron, N.; Alabouvette, C. Ribosomal DNA-Targeted Oligonucleotide Probe and PCR Assay Specific for Fusarium Oxysporum. Mycological Research 2000, 104, 518–526, doi:10.1017/S0953756299001896; [12] Liu, S.Y.; Liu, Z.; Fitt, B.D.L.; Evans, N.; Foster, S.J.; Huang, Y.J.; Latunde-Dada, A.O.; Lucas, J.A. Resistance to Leptosphaeria Maculans (Phoma Stem Canker) in Brassica Napus (Oilseed Rape) Induced by L. Biglobosa and Chemical Defence Activators in Field and Controlled Environments. Plant Pathology 2006, 55, 401–412, doi:10.1111/j.1365-3059.2006.01354.x; [13] Meincke, R.; Weinert, N.; Radl, V.; Schloter, M.; Smalla, K.; Berg, G. Development of a Molecular Approach to Describe the Composition of Trichoderma Communities. Journal of Microbiological Methods 2010, 80, 63–69, doi:10.1016/j.mimet.2009.11.001; [14] Eynck, C.; Koopmann, B.; Grunewaldt-Stoecker, G.; Karlovsky,

P.; von Tiedemann, A. Differential Interactions of Verticillium Longisporum and V. Dahliae with Brassica Napus Detected with Molecular and Histological Techniques. Eur J Plant Pathol 2007, 118, 259–274, doi:10.1007/s10658-007-9144-6.

Table S3. Mastermix composition used for the real-time PCR assays.

Target Organism(s)	DNA Polymerase	Reaction Buffer	Final MgCl2 Concentration (mM)	dNTP Concentration (µM)	Primer Concentration (µM)	Fluorescence Dye
Total fungi	Hot Start *Taq*	Standard *Taq* Buffer	2.5	100	0.3	SYBR Green®
Ascomycota	Hot Start *Taq*	Standard *Taq* Buffer	1.5	100	0.3	SYBR Green®
Basidiomycota	Hot Start *Taq*	Standard *Taq* Buffer	1.5	100	0.3	SYBR Green®
Alternaria spp.	Hot Start *Taq*	Standard *Taq* Buffer	2.0	200	0.3	SYBR Green®
Cladosporium spp.	Hot Start *Taq*	Standard *Taq* Buffer	2.0	200	0.3	SYBR Green®
Fusarium culmorum	Hot Start *Taq*	Standard *Taq* Buffer	4.0	200	0.3	SYBR Green®
Fusarium graminearum	Hot Start *Taq*	Standard *Taq* Buffer	2.5	200	0.3	SYBR Green®
Fusarium tricinctum	Hot Start *Taq*	Standard *Taq* Buffer	2.5	100	0.4	SYBR Green®
Fusarium oxysporum	Hot Start *Taq*	Standard *Taq* Buffer	2.0	200	0.4	SYBR Green®
Leptosphaeria biglobosa	*Taq*	ThermoPol® Reaction Buffer	2.0	100	0.3	SYBR Green®
Leptosphaeria maculans	*Taq*	ThermoPol® Reaction Buffer	2.0	100	0.3	SYBR Green®
Trichoderma spp.	Hot Start *Taq*	Standard *Taq* buffer	1.5	200	0.5	EvaGreen®
Verticillium longisporum	Hot Start *Taq*	Standard *Taq* buffer	3.0	200	0.3	SYBR Green®

Table S4. Thermocycling protocol used for the qPCR assays.

Target Organism(s)	Initial Denaturation	Denaturation	Annealing	Elongation	Number of Cycles
Total fungi	95°C, 120s	94 °C, 20s	55 °C, 30s	68 °C, 30s	35
Ascomycota	95°C, 120s	94 °C, 20s	55 °C, 30s	68 °C, 40s	35
Basidiomycota	95°C, 120s	94 °C, 20s	59 °C, 30s	68 °C, 40s	35
Alternaria spp.	95°C, 120s	94 °C, 10s	58 °C, 30s	68 °C, 30s	40
Cladosporium spp.	95°C, 120s	94 °C, 10s	60 °C, 30s	68 °C, 20s	40
Fusarium culmorum	95°C, 120s	94 °C, 20s	62 °C, 30s	68 °C, 45s	40
Fusarium graminearum	95°C, 120s	94 °C, 30s	61 °C, 30s	68 °C, 30s	35
Fusarium tricinctum	95°C, 120s	94 °C, 20s	65 °C, 20s	68 °C, 18s	38

Fusarium oxysporum	95°C, 120s	94 °C, 10s	68 °C, 30s [a]		40
Leptosphaeria biglobosa	95°C, 120s	94 °C, 30s	68 °C, 35s [a]		40
Leptosphaeria maculans	95°C, 120s	94 °C, 30s	68 °C, 35s [a]		40
Trichoderma spp.	95°C, 120s	94 °C, 10s	54 °C, 30s	68 °C, 35s	40
Verticillium longisporum	95°C, 120s	94 °C, 10s	60 °C, 15s	68 °C, 15s	40

For all assays, final elongation was 5 min at 68°C. [a] performed as 2-step PCR.
